# Bax Inhibitor-1 Acts as an Anti-Influenza Factor by Inhibiting ROS Mediated Cell Death and Augmenting Heme-Oxygenase 1 Expression in Influenza Virus Infected Cells

**DOI:** 10.3390/ijms19030712

**Published:** 2018-03-02

**Authors:** Mohammed Kawser Hossain, Subbroto Kumar Saha, Ahmed Abdal Dayem, Jung-Hyun Kim, Kyeongseok Kim, Gwang-Mo Yang, Hye Yeon Choi, Ssang-Goo Cho

**Affiliations:** Department of Stem Cell and Regenerative Biotechnology, Incurable Disease Animal Model & Stem Cell Institute (IDASI), Konkuk University, Seoul 05029, Korea; kawsersau07@gmail.com (M.K.H.); subbroto@konkuk.ac.kr (S.K.S.); ahmed_morsy86@yahoo.com (A.A.D.); jkim@health.southalabama.edu (J.-H.K.); proproggs@naver.com (K.K.); slayersgod@nate.com (G.-M.Y.); hyeon.choi24@gmail.com (H.Y.C.)

**Keywords:** Bax inhibitor-1, anti-influenza, reactive oxygen species, heme oxygenase-1

## Abstract

Influenza virus remains a major health concern worldwide, and there have been continuous efforts to develop effective antivirals despite the use of annual vaccination programs. The purpose of this study was to determine the anti-influenza activity of Bax inhibitor-1 (*BI-1*). Madin-Darby Canine Kidney (MDCK) cells expressing wild type *BI-1* and a non-functional *BI-1* mutant, *BI-1 ∆C* (with the C-terminal 14 amino acids deleted) were prepared and infected with A/PR/8/34 influenza virus. *BI-1* overexpression led to the suppression of virus-induced cell death and virus production compared to control Mock or *BI-1 ∆C* overexpression. In contrast to *BI-1 ∆C*-overexpressing cells, *BI-1*-overexpressing cells exhibited markedly reduced virus-induced expression of several viral genes, accompanied by a substantial decrease in ROS production. We found that treatment with a ROS scavenging agent, *N*-acetyl cysteine (NAC), led to a dramatic decrease in virus production and viral gene expression in control MDCK and *BI-1* ∆C-overexpressing cells. In contrast, NAC treatment resulted in the slight additional suppression of virus production and viral gene expression in *BI-1*-overexpressing cells but was statistically significant. Moreover, the expression of heme oxygenase-1 (*HO-1*) was also significantly increased following virus infection in *BI-1*-overexpressing cells compared to control cells. Taken together, our data suggest that BI-1 may act as an anti-influenza protein through the suppression of ROS mediated cell death and upregulation of *HO-1* expression in influenza virus infected MDCK cells.

## 1. Introduction

Influenza viruses, which are enveloped RNA viruses belonging to the family *Orthomyxoviridae*, are a significant cause of respiratory infections, causing millions of deaths annually and imposing significant economic losses [1]. Influenza virus infection is a serious health problem worldwide, resulting in morbidity and mortality in humans in the form of epidemics or pandemics [2]. Until now, annual immunization and antiviral drugs have represented the main approaches for dealing with influenza. Antiviral drugs have been proven to be effective as a preventive and therapeutic regime over the last several decades [3]. To date, three classes of anti-influenza virus drugs are available for influenza management with different modes of action: the M2 channel blockers such as amantadine and rimantadine, neuraminidase (*NA*) inhibitors (e.g., oseltamivir) [4], and RNA polymerase inhibitors (e.g., ribavirin) [5]. All of these drugs are effective against virus infection if administered quickly, but concerns have been raised because of their adverse effects and the emergence of drug-resistant influenza A/H1N1 viruses [6,7]. The development of new therapeutic agents is urgently needed to treat influenza infections.

Several novel agents that may be effective against influenza virus are currently under development. Naturally available antiviral nutrients that are usually accessible are now being used as part of the diet to combat diseases, including influenza infection [8,9]. Reactive oxygen species (ROS), which are by-products of natural metabolic pathways, can dramatically increase during periods of cellular stress, inhibiting cellular protein function and promoting cell death. Influenza virus replication in infected cells is influenced by the cellular redox status [10,11]. The inhibition of virus-induced ROS formation using different strategies, including the use of the antioxidant *N*-acetyl-l-cysteine (NAC), has been shown to inhibit influenza A virus replication [12,13,14]. Identifying cellular molecules that have activities against apoptosis and ROS will be useful to counteract influenza virus infections in cells.

Characterization of the molecular mechanisms of novel antiviral proteins is also needed to combat influenza infections, as it will aid in developing new therapeutic strategies based on the identified mechanisms. Several identified novel small molecules that have inhibited Bax [15,16] and both Bax and Bak to protect cells from apoptotic cell death [17,18]. Bax inhibitor-1 (*BI-1*) is a membrane protein containing several transmembrane domains, and it has been reported to be an anti-apoptotic protein that has a protective function against ER stress [14,19,20]. BI-1 is widely conserved in both animal and plant species. BI-1 has been reported to regulate ROS production in the ER by modifying heme oxygenase-1 (*HO-1*) expression [14,21]. Heme oxygenase-1 is an oxygenase enzyme that blocks ROS activity and is expressed to counteract ROS accumulation, thereby promoting cell survival [22,23,24,25]. *HO-1* has also been reported to act as an antiviral factor against porcine reproductive and respiratory syndrome virus infection [26] and to be induced by influenza virus infection [27]. Previous studies reported that a C-terminal mutant of *BI-1* (BI-1ΔC) relieved the effect of BI-1 overexpression regarding cell growth in transgenic mice and acted as BI-1 dominant negative mutant [19,28] and enhanced the survival and neural differentiation of embryonic stem cells by differential regulation of ROS production [29]. Thus, in this study, we determined whether *BI-1* overexpression would promote cell survival against viral infection by increasing the production of antioxidant enzymes and by destabilizing the complex responsible for ROS production, which will be helpful for the further development of novel antiviral therapeutic strategies. 

## 2. Results and Discussion

### 2.1. Overexpression of Bax Inhibitor-1 (BI-1) Relieves Virus Induced Cell Death in Madin-Darby Canine Kidney (MDCK) Cells

**Figure 1:**
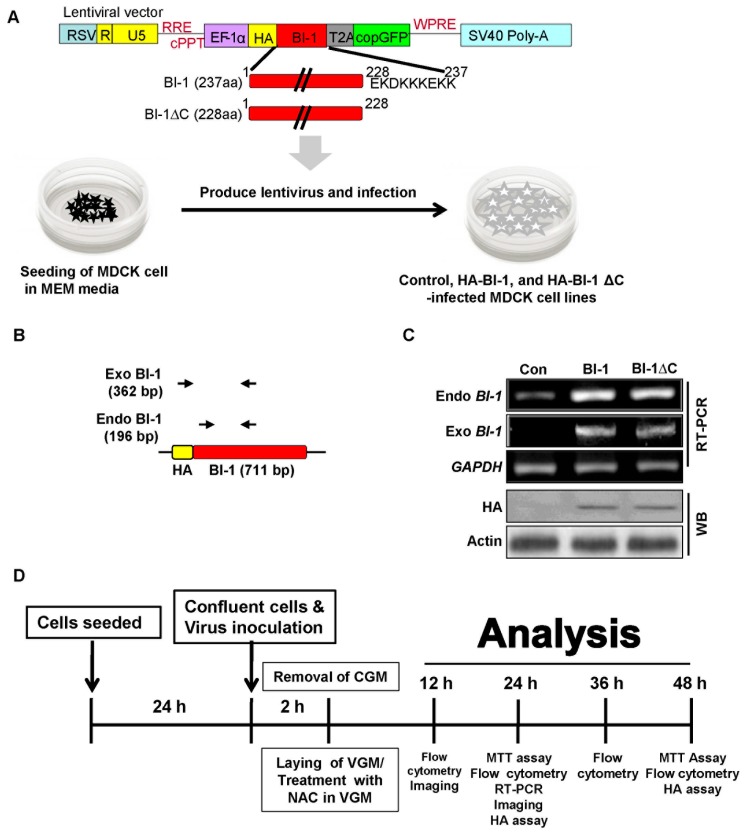
Overexpression and endogenous or exogenous expression of *BI-1* or *BI-1* ∆C in Madin-Darby Canine Kidney (MDCK) cells. (**A**) Scheme of the lentiviral constructs for the expression of wild type *BI-1* or nonfunctional mutant *BI-1* ΔC in MDCK cells. *BI-1* or *BI-1* ΔC was inserted into the lentiviral pEF vector. The pEF lentivirus containing wild type BI-1 or the non-functional mutant *BI-1* ΔC was produced and used to infect MDCK cells. (**B**) Illustration of specific primer sets which was used to analyze endogenous or exogenous expression of *BI-1*. (**C**) The endogenous or exogenous expression of BI-1 was confirmed by RT-PCR analysis in indicated cells; and the expression of *HA* was confirmed by western blot analysis in indicated cells. The expression of GAPDH and Actin was used as loading control. (**D**) The illustration of whole experimental protocol used in this study. (Abbreviations: chimeric Rous sarcoma virus (RSV) sequence, Rev response elements (RRE), central polypurine tract (cPPT), elongation factor 1 alpha (EF-1α) promoter region, a copGFP (copepod GFP) tag, a woodchuck hepatitis virus post-transcriptional regulatory element (WPRE), cells growth media (CGM), virus growth media (VGM), and hemagglutinin assay (HA assay)).

**Figure 2:**
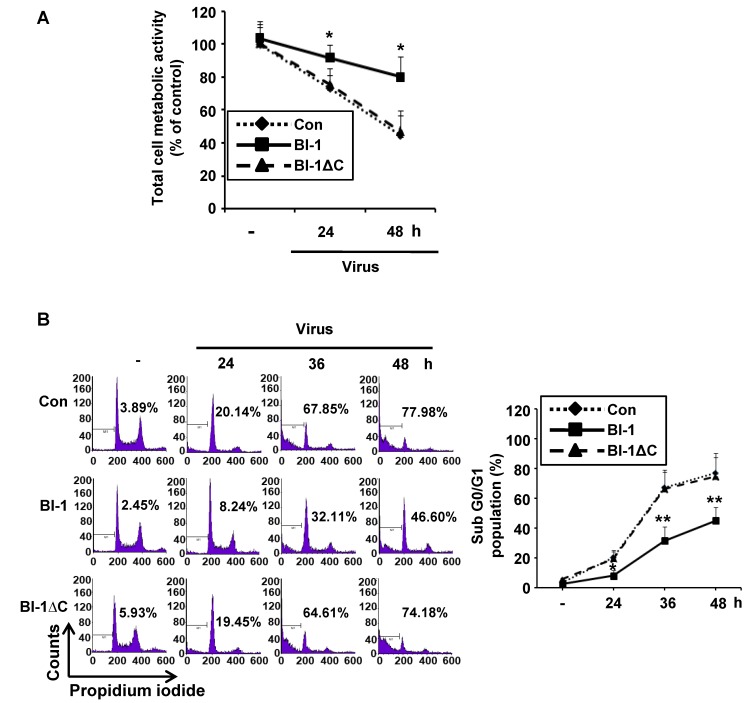
Overexpression of Bax inhibitor-1 (*BI-1*) inhibits virus induced cell death and cell cycle arrest in MDCK cells. (**A**) Control mock-infected MDCK, *BI-1*-overexpressing MDCK, and *BI-1* ΔC-overexpressing MDCK cells were seeded in 96-well cell culture plates and infected with A/PR/8/34 virus at 100 TCID_50_. The media were changed 2 h after virus infection, and MTT assays were performed 24 h and 48 h after virus infection. Data were shown as the relative expression of cell metabolic activity of *BI-1*-overexpressing MDCK, and *BI-1* ΔC-overexpressing MDCK cells compared to the control infection from three individual experiments (mean ± SEM) (* *p* < 0.05). (**B**) Control MDCK, *BI-1*-overexpressing MDCK, and *BI-1* ΔC-overexpressing MDCK cells were seeded in 6-well cell culture plates and infected with A/PR/8/34 virus at 1000 TCID_50_. Anti-influenza activity was determined three times calculating the apoptotic sub-G0/G1 populations by flow cytometric analysis 24 h, 36 h, and 48 h after virus infection. Data were expressed as relative numbers of apoptotic sub-G0/G1 populations of *BI-1*-overexpressing MDCK, and *BI-1* ΔC-overexpressing MDCK cells compared to the control (mean ± SEM) (* *p* < 0.05, ** *p* < 0.01).

After preparing Madin-Darby Canine Kidney (MDCK) cells expressing the lentiviral construct containing wild type Bax inhibitor-1 (*BI-1*) or the non-functional deletion mutant form (BI-1 ∆C; deletion of 9 amino acids (EKDKKKEKK) from the C-terminal region of BI-1) (Figure 1A) [19,29], we confirmed the endogenous or exogenous expression of *BI-1* from the control, wild type BI-1, and non-functional *BI-1* ∆C overexpressing MDCK cells by RT-PCR analysis using endogenous or exogenous specific primer set and overexpression was also confirmed by western blot analysis (Figure 1B,C). Then, the *BI-1* or *BI-1* ∆C-overexpressing MDCK cells were used in an antiviral experiment against influenza virus A/PR/8/34, as illustrated in Figure 1D. First, the anti-influenza activity of *BI-1* overexpressing cells was determined using cell viability assays after influenza virus administration, revealing that the overexpression of BI-1 in MDCK cells led to the significant suppression of virus-induced cell death compared to that in mock or *BI-1* ∆C-overexpressing cells (Figure 2A). Flow cytometric analysis of the apoptotic sub G0/G1 populations also confirmed the significant anti-influenza virus activity of *BI-1* (Figure 2B), which supported our previous report that C-terminal amino acid deletion of *BI-1* acted in a dominant negative fashion [19,29].

### 2.2. Overexpression of BI-1 Inhibits Viral Replication and Viral Gene Expression in MDCK Cells

**Figure 3:**
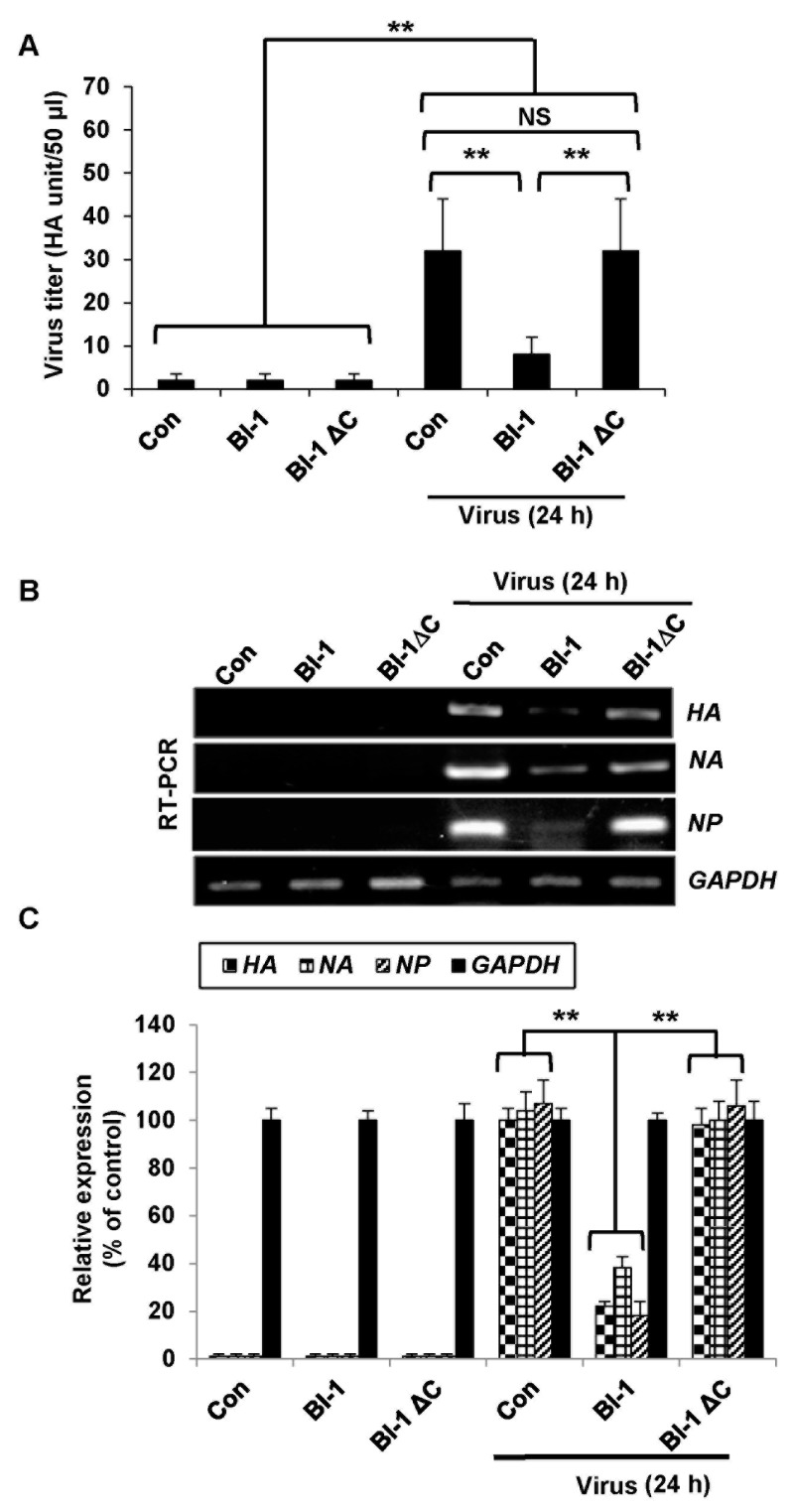
Overexpression of *BI-1* impedes viral replication and viral gene expression in MDCK cells. (**A**) Control mock MDCK, *BI-1*-overexpressing MDCK, or *BI-1* ΔC-overexpressing MDCK cells were infected with A/PR/8/34 virus at 1000 TCID_50_, and hemagglutination activity was measured as described in the materials and methods. *HA* titers were calculated triplicates as hemagglutination units/50 µL (HAU/50 µL) (** *p* < 0.01). (**B**) *BI-1*- or *BI-1* ΔC-overexpressing MDCK cells cultured in 6-well cell culture plates were infected with influenza A/PR/8/34 virus at 1000 TCID_50_, and RT-PCR was performed to determine the expression of the viral *HA*, *NA*, and *NP* genes. (**C**) Relative expression of the *HA*, *NA*, and *NP* genes was analyzed thrice by real time qRT-PCR and presented as a percentage of the control (** *p* < 0.01). (Abbreviations: hemagglutinin (*HA*), neuraminidase (*NA*), and nucleoprotein (*NP*)).

To further analyze the antiviral activity of BI-1 on influenza virus production, we conducted a hemagglutination assay [30] using red blood cells (RBCs). We determined whether overexpression of BI-1 would interfere with viral adsorption to RBCs (Figure 3A). A virus yield reduction *HA* assay with the media produced in the culture of the virus-infected *BI-1*- or *BI-1* ∆C-overexpressing MDCK cells showed that BI-1 overexpression, but not *BI-1* ∆C overexpression, led to a substantial reduction in virus yield compared to the control. More than a four-fold reduction in virus production was observed in *BI-1*-overexpressing cells compared to that in the mock control or *BI-1* ∆C-overexpressing cells. RT-PCR analysis of hemagglutinin (*HA*), neuraminidase (NA), and nucleoprotein (NP) gene expression revealed that the expression of these viral genes was significantly suppressed with *BI-1* overexpression but not mock control and *BI-1* ∆C overexpression (Figure 3B,C).

### 2.3. BI-1 Overexpression Suppresses ROS-Mediated Influenza Virus Infection in MDCK Cells

**Figure 4:**
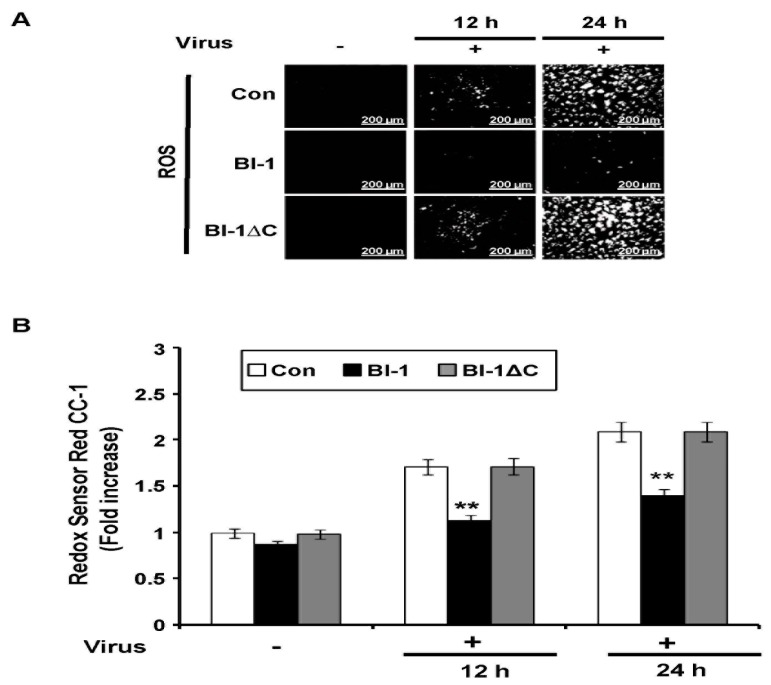
BI-1 overexpression regulated ROS production in influenza virus-infected MDCK cells. (**A**) Control mock-MDCK, *BI-1*-overexpressing MDCK, and *BI-1*ΔC-overexpressing MDCK cells were seeded in 6-well plates and infected with A/PR/8/34 virus at 100 TCID_50_. Intracellular ROS levels were measured using Redox Red sensor CC-1. Fluorescence-stained cells were imaged on an inverted fluorescence microscope. (Scale bar- 200 µm). (**B**) Relative expression of ROS was analyzed thrice by flow cytometer in indicated virus-infected cells (** *p* < 0.01).

As influenza virus infection modulates ROS production in infected cells [12,13], we evaluated whether *BI-1* overexpression provided protection against virus-induced ROS production. Virus-induced ROS production was significantly suppressed by *BI-1* overexpression, but not by *BI-1* ∆C overexpression (Figure 4A). These observations were confirmed by flow cytometric analysis of the cells stained with Redox Red sensor CC-1 (Figure 4B). In the virus-infected mock control and *BI-1* ∆C-overexpressing cells, ROS production obviously increased following virus infection, but the virus-induced ROS production was significantly suppressed in the *BI-1*-overexpressing MDCK cells. These data indicated that *BI-1* would act as anti-influenza virus factor by protecting cells against virus-induced ROS production and apoptosis.

### 2.4. BI-1 May Independently Act as an Antiviral Protein through the Suppression of ROS Production by Activating the Expression of HO-1 with Additional Effect of the ROS Scavenging Agent NAC

**Figure 5:**
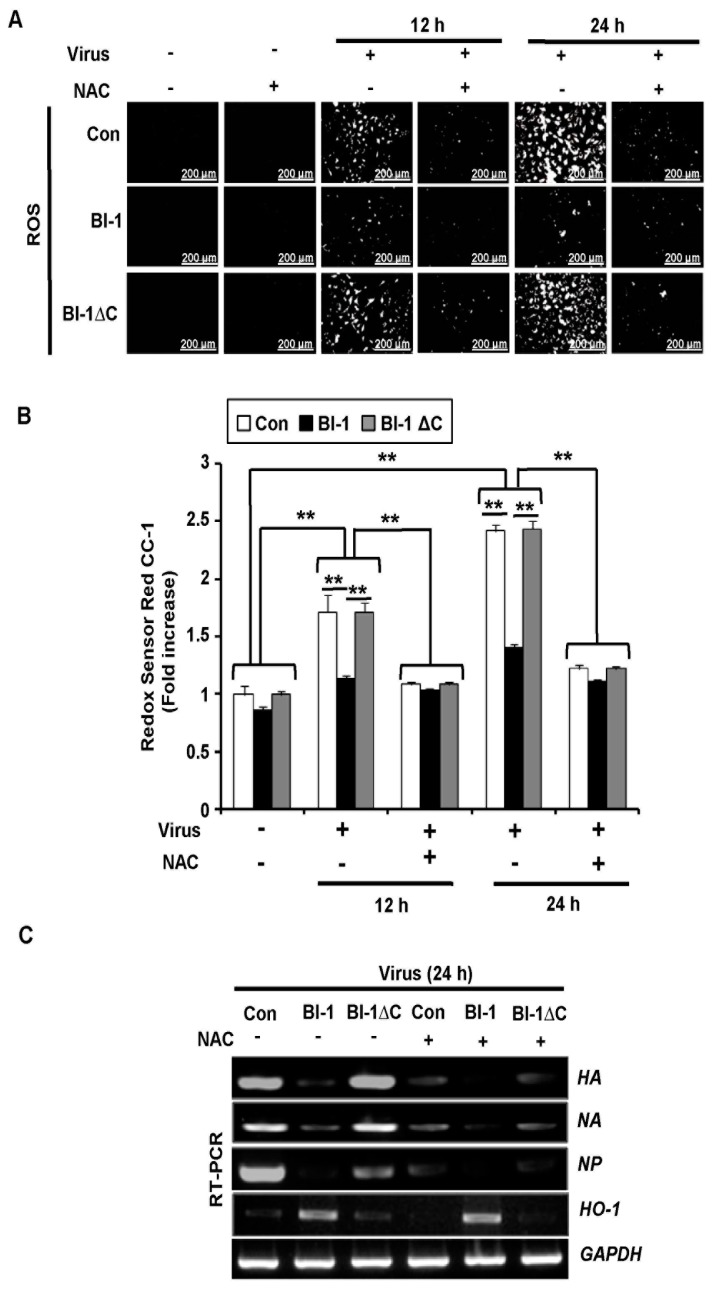

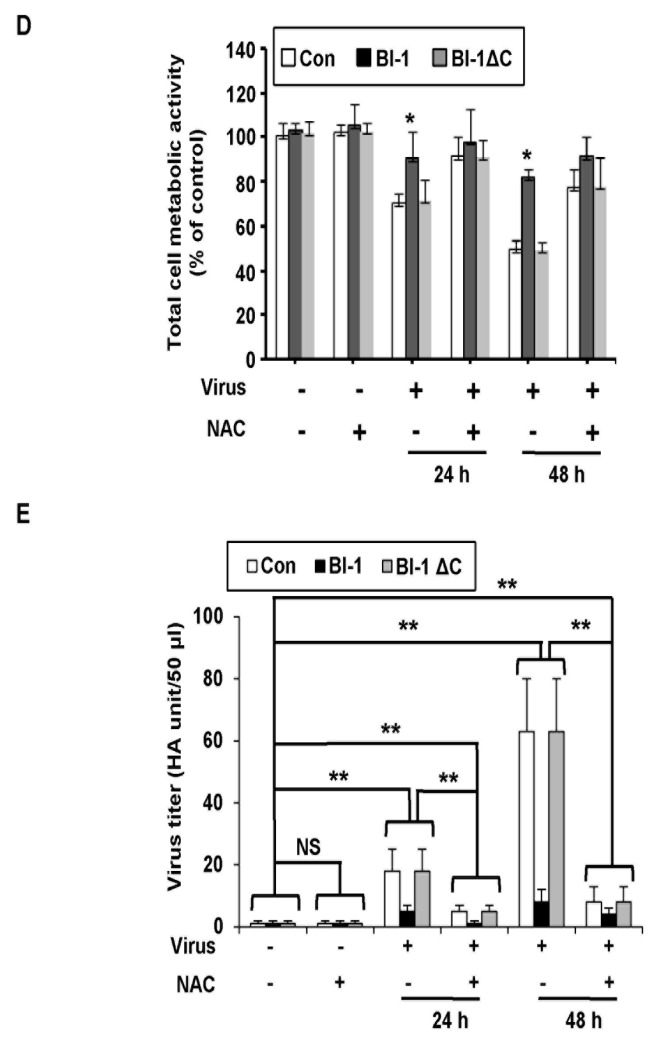
*BI-1* may act as an antiviral protein through the suppression of ROS production and ROS-independent regulation of *HO-1* expression. (**A**) Control mock MDCK, *BI-1*-overexpressing MDCK, or *BI-1* ΔC-overexpressing cells were infected with A/PR/8/34 virus at 1000 TCID_50_ and treated with NAC as an antioxidant agent. Intracellular ROS levels were measured in control mock MDCK, *BI-1*-overexpressing MDCK, and *BI-1* ΔC-overexpressing MDCK cells incubated with Redox Red sensor CC-1. Fluorescence-stained cells were imaged on an inverted fluorescence microscope. (Scale bar- 200 µm). (**B**) Relative expression of ROS was analyzed thrice by flow cytometer in indicated virus-infected cells with or without NAC treatment (** *p* < 0.01). (**C**) RT-PCR analysis of virus-infected cells to determine the expression of viral genes such as *HA*, *NA*, and *NP* and the endogenous *HO-1* gene in the host cells. (**D**) Cells were infected with A/PR/8/34 virus at 100 TCID_50_ and treated with NAC. MTT assay was performed 24 h and 48 h after virus infection, and the relative expression compared to the control infection results are shown as the mean ± SEM from three individual experiments (* *p* < 0.05). (**E**) Hemagglutination assay of the virus-infected cells with and without NAC treatment. *HA* titers were calculated triplicates as hemagglutination units/50 µL (HAU/50 µL) using 1% chicken RBCs (** *p* < 0.01). (Abbreviations: hemagglutinin (*HA*), neuraminidase (*NA*), nucleoprotein (*NP*), and heme oxygenase-1 (*HO-1*)).

Next, we treated the virus-infected cells with N-acetyl cysteine (NAC) to investigate the effect of ROS on the antiviral function of *BI-1*. NAC treatment led to the significant suppression of virus-induced ROS production in the mock control and *BI-1* ∆C-overexpressing MDCK cells (Figure 5A). The additional suppression of ROS production was also observed by *BI-1* overexpressing cells following NAC treatment. These observations were then confirmed by flow cytometric analysis of the intracellular ROS production (Figure 5B). In the virus-infected mock MDCK and *BI-1* ∆C-overexpressing cells, ROS production was significantly increased about two-fold compared to the uninfected cells, while *BI-1* overexpression led to impede the suppression of ROS production. The treatment of NAC in virus treated cells led to a significant reduction in ROS production in mock control and BI-1 ∆C-overexpressing MDCK cells, while *BI-1* overexpressing MDCK cells became like untreated cells after 12 h (Figure 5A,B). After 24 h of virus treatment, ROS level was augmented dramatically in both control and *BI-1* ∆C-overexpressing MDCK cells which could be diminished by NAC treatment (Figure 5A,B). In addition, *BI-1* overexpressing MDCK cells showed a reduction in virus induced ROS level and the reduction was slightly more but statistically significant in NAC treatment group (Figure 5A,B). The expression of viral genes, such as *HA*, *NA*, or *NP*, was also significantly inhibited by *BI-1* overexpression, and the additional reduction of the viral genes expression was also detected by NAC treatment (Figure 5C). Of note, the expression of heme oxygenase-1 (*HO-1*), which counteracts the accumulation of ROS in stressed cells, was significantly induced following *BI-1* overexpression, but the enhanced *HO-1* expression in BI-1-overexpressing cells was not suppressed following NAC treatment, suggesting that the *BI-1*-induced enhancement of *HO-1* expression may play an important role in the *BI-1* overexpression-induced antiviral activity, probably in a ROS-independent manner which need to be studied in detail. The cell viability and virus titer production results also showed that *BI-1* may act as an antiviral protein through the suppression of ROS production and ROS-independent regulation of *HO-1* expression in addition to the effect of the ROS scavenging agent NAC (Figure 5D,E).

**Figure 6:**
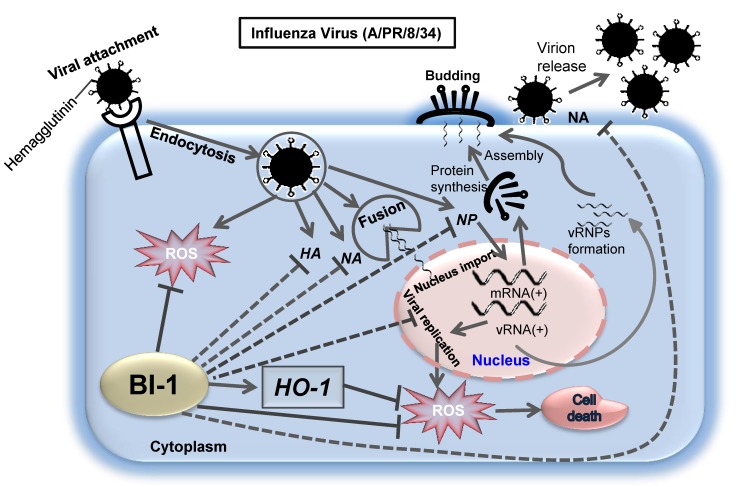
Mechanism of action of Bax inhibitor-1 to protect MDCK cells from influenza virus infection-induced ROS production and cell death. After attachment to the host cell, the influenza virus induces viral replication and ROS production in the host cells. *BI-1* overexpression in influenza virus-infected cells enhances *HO-1* expression and suppresses ROS production, accompanied by a reduction in viral replication and propagation. BI-1 may inhibit the new virion assembly and release from the infected cells by inhibiting function of viral genes including *HA*, *NA*, and *NP*. (Abbreviations: hemagglutinin (*HA*), neuraminidase (*NA*), nucleoprotein (*NP*), viral ribonucleoproteins (vRNPs), and heme oxygenase-1 (*HO-1*)).

The emergence of new strains influenza virus and influenza infections with high morbidity and mortality have been reported in humans, which have a pandemic threat. Although vaccines are used to prevent influenza infections, they take time to impart protection but the viruses may escape immunity induced by vaccination due to antigenic discrepancy between the vaccine strain and the circulating strain or due to antigenic drift [6,7]. Therefore, developing effective antiviral drugs is urgently needed to combat the disease. Influenza virus infection of susceptible host cell causes virus propagation, cell death, and viral gene expression. Notably, following influenza virus infection in the MDCK cell line, the increase in the titer of the influenza virus increases following ROS production [12]. In our previous studies, we found that influenza virus infection, replication, and propagation were prevented by several natural products [31,32]. In this study, we investigated the cell membrane protein BI-1 and found that anti-apoptotic protein *BI-1* regulated the cellular responses to influenza virus infection. Importantly, the overexpression of *BI-1* in MDCK cells impaired influenza virus production, propagation, and the synthesis of viral genes. *BI-1* prevented virus adsorption into the MDCK cells by interfering with viral membrane fusion to the cells. *BI-1* overexpression also suppressed *NA* activity to inhibit virion release, viral RNA synthesis by inhibiting *NP*, and virus titer production in MDCK cells. Previous studies have reported that *BI-1* functions as an anti-apoptotic protein and ROS scavenger [14,20,22], recently in another study we had reported that in human embryonic kidney cells, *BI-1* overexpression significantly suppressed ROS production, while *BI-1*ΔC did not make any significant effect on ROS production [19], indicating that the C-terminal mutant plays in dominant negative fashion. In our studies, we found that the overexpression of *BI-1* significantly reduced apoptotic cell death and ROS production in influenza-infected MDCK cells. Thus, *BI-1* overexpression in MDCK cells suppressed ROS production and thereby inhibited viral gene and viral titer production, propagation, and cytopathogenicity (Figure 6). Moreover, our study revealed that *BI-1*-overexpressing MDCK cells were more resistant to influenza virus infection and virus-induced cell death.

The anti-oxidant molecule NAC is known to inhibit ROS production, viral replication, and virus-induced apoptosis in influenza-infected cells [33]. As *BI-1* had shown a significant anti-influenza effect against A/PR/8/34 including substantial reduction of ROS production, in our studies we used NAC as ROS scavenger whether it had any notable effect in *BI-1* overexpressing cell. In this study, in *BI-1*-overexpressing MDCK cells, NAC had an additional effect on reducing ROS and viral replication but it was not significant, although, in the mock control and *BI-1* ∆C overexpressing cells, it had a significant effect on reducing ROS and inhibiting virus-induced cell death and viral replication and propagation. Moreover, in our studies, we had observed activated expression *HO-1* in influenza infected *BI-1* overexpressing cells. Although ROS production was significantly reduced but, the *BI-1*-induced increase in *HO-1* expression was not suppressed by NAC treatment, possibly because of the enhanced expression of HO-1 protein in *BI-1*-overexpressing cells and because *HO-1* may play an important role in the suppression of virus-induced ROS production, cell death, virion release from host cells, and the replication and propagation of the viral *HA, NA*, and *NP* genes.

BI-1 has been shown to regulate ROS accumulation during cellular stress [22,30,34,35], and here, we present evidence that influenza virus-induced ROS accumulation was suppressed following *BI-1* overexpression. *HO-1* is known to be activated during cellular stress and influence pro-oxidant signals such as ROS. Interestingly, in our study, we found that *HO-1*, which is a ROS scavenger, was strongly upregulated in *BI-1*-overexpressing cells. Thus, highly activated cellular *HO-1* in BI-1-overexpressing MDCK cells reduced ROS accumulation following influenza infection. *HO-1* may be important for the cytoprotective function of *BI-1* as *BI-1* overexpressing cells, which had a higher basal level of *HO-1*, had a survival advantage against influenza virus infection.

Taken together, our findings indicate that the overexpression of *BI-1* in the MDCK cell line played a key role in the regulation of host cellular defense mechanisms against influenza virus infection. The overexpression of *BI-1* slowed down the ROS-dependent propagation of influenza virus and virus-induced pathogenicity and induced increased expression of *HO-1*. Our study adds to previous efforts to identify small molecules for use against influenza infection, and our results regarding the anti-apoptotic protein *BI-1* as an anti-influenza agent that can suppress ROS production will facilitate the development of novel mechanism-based therapeutics against influenza virus infection, but further studies are needed to elucidate the mechanisms involved.

## 3. Materials and Methods

### 3.1. Cells, Virus, Reagents, and Lentiviral Infection

Madin Darby Canine Kidney (MDCK) cells were obtained from the American Type Culture Collection (ATCC CCL-3, Manassas, VA, USA) and maintained in a minimum essential medium (MEM) (Gibco, Carlsbad, CA, USA) supplemented with 10% fetal bovine serum (FBS), and 100 U/mL penicillin/streptomycin (Gibco). Influenza virus A Puerto Rico/8/34 was kindly provided by the Immunogenetics Lab., Department of Animal Biotechnology, Konkuk University. MDCK cells were washed with PBS before virus infection and cultured in virus growth medium (MEM without FBS) supplemented with 10% bovine serum albumin, 100 U/mL penicillin/streptomycin, and 2 µg/mL trypsin TPCK (Sigma, St. Louis, MO, USA).

It is known that MDCK cells are the best choice to study the influenza virus infection due to rapid virus growth capability and great adaptation in serum free media [36]. BI-1 is naturally present in cells to protect cellular death. According to the result of amino acid sequence alignment, more than 97% of amino acid sequence was same in both human and canine BI-1 protein. In this study, our main purpose is to find the role of *BI-1* in influenza virus induced cell death. Thus, we used our available human BI-1 lentivirus expressing construct [19]. Analysis of the amino acid sequences of BI-1 protein was performed using data obtained from Gen Bank (NCBI). The accession number of the protein sequence of *BI-1* was NP_003208.1. Scan Prosite (http:www.expasy.org/tools/scanprosite/PROSITE) and NCBI RPS-BLAST (http://www.ncbi.nlm.nih.gov/Structure/cdd/wrpsb.cgi) were used for conserved domain database screening. The lentiviral construct containing wild type Bax inhibitor-1 (*BI-1*) or the non-functional deletion mutant form (BI-1 ∆C; deletion of 9 amino acids (EKDKKKEKK) from the C-terminal region of BI-1) was prepared, and the lentiviral vector particles were purified from the supernatant of transiently transfected 293T cells. The cell supernatant was collected 72 h post-transfection, filtered through polyether sulfone membranes (0.45-μm pore size), and concentrated 120-fold by ultracentrifugation (50,000× *g* for 90 min at 4 °C). The pellet was resuspended in the cell culture medium and subsequently added to the cells. After 1 week of culture, the cells were centrifuged and resuspended in PBS and sorted with a FACSVantage™ flow cytometer (Becton Dickinson, Franklin Lakes, NJ, USA). The sorted MDCK cells were cultured in virus growth medium (MEM without FBS) supplemented with 10% bovine serum albumin, 100 U/mL penicillin/streptomycin, and 2 µg/mL trypsin TPCK. 

For lentiviral transfection, MDCK cells were incubated overnight at a density of 2 × 10^6^ cells per 60-mm culture dish in MEM medium (Gibco, Carlsbad, CA, USA) supplemented with 10% FBS and 100 U/mL penicillin/streptomycin (Gibco) and then infected with the indicated expression vectors using the lentivirus vector. For the selection of MDCK cells that stably expressed wild type *BI-1* or the non-functional mutant *BI-1* ΔC (with the C-terminal 14 amino acids deleted), cells were transfected with 6 μg pEF *HA* (control), pEF HA-BI-1, or BI-1ΔC using a central polypurine tract (cPPT), promoter region, and a copGFP (copepod GFP) tag. After selection, surviving MDCK cells were analyzed by RT-PCR using the following primers: BI-1 (F): 5′-GCAGGGGCCTATGTCCAT-3′ BI-1 (R): 5′-AGGATGCTGGGGTTGACAGC-3′, and HA-Exo (F): 5′-TACGATGTTCCAGATTACGCT-3′. A mutant form (*BI-1* ΔC) of *BI-1* was constructed by deletion of the last nine amino acids at the protein’s C-terminal region [19,29]. The primers for the C-terminal truncation of *BI-1* were *BI-1* ΔC (F) 5′-GGGAAGAATTCATGAACATATTTGATCGA-3′ and (R) 5′-GGGAACTCGAGTCACTAGGA -CCGGTACTTA-3′.

### 3.2. Cell Viability and Cell Cycle Analysis 

The mock MDCK, BI-1-overexpressing MDCK, and BI-1 ∆C-overexpressing MDCK cells were seeded in 96-well plates for the measurement of cell viability at a concentration of 2 × 10^4^ cells per well. Twenty-four hours after seeding, when the cells were confluent, they were washed twice with PBS and infected with the virus at 100 TCID_50_ and incubated at 37 °C for 2 h in a 5% CO_2_ incubator. The virus-containing media were replaced with virus growth media 2 h after infection. MTT assays (Sigma-Aldrich, St. Louis, MI, USA) were performed 24 and 48 h after infection according to the manufacturer’s instructions using a spectrophotometer (Bio-Rad, Hercules, CA, USA) at a wavelength of 490 nm to determine the total cell metabolic activity. For cell cycle analysis, mock, *BI-1*-overexpressing, and *BI-1* ∆C-overexpressing MDCK cells were harvested in 24, 36, and 48 h time frame after virus infection by trypsin-EDTA (Gibco), and stained with propidium iodide (PI) (Sigma-Aldrich) following manufacturer protocol and analyzed by a Beckman Coulter FC-500 flow cytometer (Beckman Coulter, Fullerton, CA, USA) using Cell Quest 3.2 software. The percentage of the apoptotic sub-G0/G1 populations of *BI-1*-overexpressing MDCK, and BI-1 ΔC-overexpressing MDCK cells were counted and compared to the control.

### 3.3. Virus Yield Reduction Assay 

The mock control cells, overexpressing MDCK-BI-1, and *BI-1* ∆C-overexpressing MDCK cells were seeded in 6-well cell culture plates at a concentration of 2–3 × 10^5^ cells per well. When the cells were confluent, the cell growth media were removed, and the cells were washed twice with PBS and infected with the virus at 1000 TCID_50_ diluted in virus-containing media. The virus-containing media were replaced by virus growth media, and HA assays were performed 48 h after virus infection. Briefly, 25 μL of PBS was added to each well of the 96-well round bottom plates. Then, 25 μL of the cell supernatant containing the virus was diluted by two-fold serial dilution in each well, and 25 μL of PBS was added again to each well. Then, 50 μL of 1% chicken RBCs was added to each well followed by incubation for 1 h at room temperature to observe hemagglutination activity. HA titers were calculated as hemagglutination units/50 µL (HAU/50 µL).

### 3.4. Intracellular ROS Detection 

The mock MDCK, MDCK-*BI-1* overexpressing, and MDCK-*BI-1* ∆C overexpressing cells were seeded at a concentration of 2–3 × 10^5^ cells per well in 6-well cell culture plates. The media were removed when the cells were confluent, and the cells were washed twice with PBS and infected with the virus at 1000 TCID_50_ in virus-containing media. Then, 12 h, 24 h and 48 h after virus infection, the cells were exposed to Redox Red sensor CC-1 (Molecular Probes, Inc., Eugene, OR, USA) for 30 min in the dark according to the manufacturer’s instructions to determine ROS levels. The cells were then washed and trypsinized, and the fluorescence intensity was quantified and analyzed with a Beckman Coulter FC-500 flow cytometer (Beckman Coulter, Fullerton, CA, USA) using Cell Quest 3.2 software. At least 10,000 cells were analyzed from each sample. For the measurement of intracellular ROS levels, fluorescence-stained cells were imaged with an inverted fluorescence microscope. To determine ROS scavenging activity, 2 h after virus infection, the cells were treated with 10 mM NAC. Intracellular ROS levels were measured in control MDCK, *BI-1*-overexpressing MDCK, and *BI-1* ΔC-overexpressing MDCK cells using Redox Red sensor CC-1. Fluorescence-stained cells were imaged with an inverted fluorescence microscope to determine the intensity of ROS production, and flow cytometry was performed for ROS quantification.

### 3.5. RT-PCR

Confluent mock MDCK, *BI-1*-overexpressing MDCK, and *BI-1*∆C-overexpressing MDCK cells grown in 6-well plates were infected with influenza A/PR/8/34 virus at 100 TCID50. The cells were scraped off 24 h after infection, and cell pellets were collected by centrifugation (1500× *g* for 5 min). Cell pellets were washed two times with PBS, and total cellular and viral RNAs were isolated using Easy Blue total RNA extraction kits (Intron Biotechnology). First-strand cDNA was synthesized from 3 µg of total RNA. PCR reactions were performed using primers for *HA*, (F): 5’-GAAAGCTCATGGCCCAACCA-3’ and (R): 5’-TCCCAGGGGTGTTTGACACT-3’; *NA*, (F): 5’-TGCTTGGTCAGCAAGTGCAT-3’ and (R): 5’-GGTTGTCACCGAAAACCCCA-3’; *NP*, (F): 5’-TGCTTCAAAACAGCCAAGTG-3’ and (R): 5’-GCCCAGTACCTGCTTCTCAG-3’; and *HO-1*, (F): 5’-ACATCTATGTGGCCCTGGAG-3’ and (R): 5’-CCTGCAACTCCTCAAAGAGC-3’. The amplification conditions were as follows: 95 °C for 10 min, 95 °C for 30 s, 55 °C for 30 s, and 72 °C for 1 min, 72 °C, 4 °C (30 cycles). The expression of the cellular housekeeping gene *GADPH* was used as a control to determine the amount of RNA in the assay.

### 3.6. Western Blot Analysis

The MDCK, over expressed MDCK-BI-1 and over expressed MDCK-BI-1∆C cells seeded in 60 mm dishes and infected with influenza A/PR/8/34 virus at 1000 TCID_50_, were washed 3 times in ice-cold PBS, scraped from the dishes, and collected in extraction buffer (1% TritonX-100, 100 mM Tris–HCl, pH 7.5, 10 mM NaCl, 10% glycerol, 50 mM sodium fluoride, and 1 mM phenylmethylsulfonyl fluoride (PMSF)). After the cells had been incubated on ice for 30 min, the lysates were centrifuged and protein containing cleared lysates was quantified using the Bradford Protein Assay Reagent (Bio-Rad). Equal amounts of protein were then separated on 10–12% SDS PAGE gels and electrophoretically transferred on to nitrocellulose membranes (0.2 mm). These membranes were blocked with 3–5% non-fat dry milk and Tris-buffered saline (TBS) and subsequently probed for *HA* and Actin in TBS containing 3% non-fat dry milk. Antibody–antigen complexes were detected using goat anti-mouse IgG or goat anti-rabbit IgG peroxidase conjugates, followed by use of an enhanced chemiluminescence (ECL) detection kit from Amersham (Buckinghamshire, UK).

### 3.7. Statistical Analysis

The results were expressed as the mean ± SE. Each value is the mean of at least three independent experiments in each group. The statistical significance of the differences between the two cell populations was determined using the two-tailed Student’s *t*-test (Origin), and *p* values equal to or less than 0.05 and 0.01 were considered significant.

## Abbreviations

BI-1	Bax inhibitor-1
ERK	Extracellular Signal–regulated kinase
STAT	Signal transducer and activator of transcription
NAC	*N*-acetyl cysteine
ROS	Reactive oxygen species
HO-1	Heme oxygenase-1
HA	Hemagglutinin
NA	Neuraminidase
M2	Matrix-2
MDCK	Madin Darby Canine Kidney
cPPT	Central polypurine tract
TCID_50_	Tissue culture infective dose 50
PBS	Phosphate buffered saline
IgG	Immunoglobulin G
ECL	Enhanced chemiluminescence
FBS	Fetal bovine serum
GFP	Green fluorescent protein
WPRE	Woodchuck hepatitis virus post-transcriptional regulatory element
